# Microbial Interactions in the Phyllosphere Increase Plant Performance under Herbivore Biotic Stress

**DOI:** 10.3389/fmicb.2017.00041

**Published:** 2017-01-20

**Authors:** Muhammad Saleem, Nicole Meckes, Zahida H. Pervaiz, Milton B. Traw

**Affiliations:** ^1^Department of Biological Sciences, University of Pittsburgh, PittsburghPA, USA; ^2^Department of Biology, Berea College, BereaKY, USA

**Keywords:** phyllosphere, plant–microbe–insect interactions, beneficial and pathogenic bacteria, plant performance, biotic stress, herbivory, antagonistic interactions, bacterial species richness

## Abstract

The phyllosphere supports a tremendous diversity of microbes and other organisms. However, little is known about the colonization and survival of pathogenic and beneficial bacteria alone or together in the phyllosphere across the whole plant life-cycle under herbivory, which hinders our ability to understand the role of phyllosphere bacteria on plant performance. We addressed these questions in experiments using four genetically and biogeographically diverse accessions of *Arabidopsis thaliana*, three ecologically important bacterial strains (*Pseudomonas syringae* DC3000, *Xanthomonas campestris*, both pathogens, and *Bacillus cereus*, plant beneficial) under common garden conditions that included fungus gnats (*Bradysia* spp.). Plants supported greater abundance of *B. cereus* over either pathogenic strain in the phyllosphere under such greenhouse conditions. However, the Arabidopsis accessions performed much better (i.e., early flowering, biomass, siliques, and seeds per plant) in the presence of pathogenic bacteria rather than in the presence of the plant beneficial *B. cereus*. As a group, the plants inoculated with any of the three bacteria (*Pst DC3000, Xanthomonas*, or *Bacillus*) all had a higher fitness than uninoculated controls under these conditions. These results suggest that the plants grown under the pressure of different natural enemies, such as pathogens and an herbivore together perform relatively better, probably because natural enemies induce host defense against each other. However, in general, a positive impact of *Bacillus* on plant performance under herbivory may be due to its plant-beneficial properties. In contrast, bacterial species in the mixture (all three together) performed poorer than as monocultures in their total abundance and host plant growth promotion, possibly due to negative interspecific interactions among the bacteria. However, bacterial species richness linearly promoted seed production in the host plants under these conditions, suggesting that natural enemies diversity may be beneficial from the host perspective. Collectively, these results highlight the importance of bacterial community composition on plant performance and bacterial abundance in the phyllosphere.

## Introduction

Plants and insects have evolved together for more than 400 million years, with the phyllosphere as an important center of their intimate and complex interactions. Over these eons of time, both have developed complex interactions with microbes. These plant–microbe and insect–microbe interactions significantly influence plant performance and defense (Sugio et al., 2014). Some studies have revealed that microbes (pathogenic or beneficial) and insect herbivores could potentially determine the distribution, abundance, and diversity of plant species in particular communities (Bell et al., 2006; Bagchi et al., 2010, 2014). Although these plant–microbe–insect (PMI) interactions are ubiquitous in nature, most prior research has focused separately on one aspect only, either the plant-microbe or the plant-insect dynamic (Tack and Dicke, 2013). To bridge this gap, recently there has been a surge of interest in studying the PMI interactions, as is emphasized in several recent reviews (Pieterse and Dicke, 2007; Pieterse et al., 2012; Biere and Bennett, 2013).

There have been some integrated studies mainly examining phytohormone signaling (Stout et al., 2006; Pieterse and Dicke, 2007; Schenk et al., 2008), fungal pathogenicity (Cardoza and Tumlinson, 2006; Biere and Bennett, 2013; Tack and Dicke, 2013), and rhizobacteria-mediated biological control of insect pests (Sugio et al., 2014). Overall, these studies on the PMI interactions have reported that plants infected with pathogens may have a negative, positive, or no effect on insect herbivory; particularly with respect to systems with fungal pathogens, insects, and plants (Tack and Dicke, 2013).

The studies investigating the significance of native phyllosphere pathogenic or beneficial bacteria on plant performance under herbivory are few (but see, Humphrey et al., 2014). Stout et al. (1999) showed that the leaf chewing caterpillar (Lepidoptera; Noctuidae) and corn earworm (*Helicoverpa zea*) exhibited reduced growth while feeding on the tomato leaves infected with *Pseudomonas syringae* pv. *tomato DC3000* (*Pst DC3000*). Moreover, Cardoza and Tumlinson (2006) reported reduced herbivory of armyworm on the pepper leaves infected with the *Xanthomonas campestris* pv. *armoraciae*. Similarly, Van Oosten et al. (2008) revealed that Arabidopsis leaves treated with *Pseudomonas fluorescens WCS417r* and *Pst DC3000* showed increased resistance against generalist herbivores. Conversely, Vos et al. (2006) demonstrated that the caterpillar feeding (*Pieris rapae*) significantly decreased the diseases caused by the *Pst DC3000* and *Xanthomonas*, but this effect was observed only in the caterpillar-damaged local leaf tissues. In some other short-term studies, the application of bacteria (*Xanthomonas* and *Pst DC3000*) on mature leaves influenced flowering phenology, plant architecture and reproductive output (e.g., Kover and Schaal, 2002; Korves and Bergelson, 2003). Most of the previous studies investigated PMI interactions at the individual leaf level in experiments where the selected leaves were exposed individually to the larval herbivory in petri plates or small cages over short timescales (Stout et al., 1999; Cardoza and Tumlinson, 2006; Vos et al., 2006; Van Oosten et al., 2008). In contrast, studies investigating PMI interactions over the longer timescales are scarce, which limits our ability to comprehend and generalize their impacts on complex assemblages of species.

At present, relatively less is understood about the effects of phyllosphere beneficial or pathogenic bacteria or their mixtures on plant performance (see, Traw et al., 2007; Saleem, 2015a), especially under conditions that include the presence of insect herbivores and the resulting possibility of tritrophic interactions (Humphrey et al., 2014; Saleem and Moe, 2014; Sugio et al., 2014; Saleem, 2015b). Such studies investigating tritrophic interactions involving ecologically diverse plants and microbes are important in part because colonization of the phyllosphere by bacteria may be accelerated under insect herbivory (Humphrey et al., 2014). Thus, it is an interesting avenue to see whether and how various bacterial species interact with each other and host plants, and what could be the impact of these interactions on plant-microbe performance. Here, we studied colonization of the phyllosphere by both types of bacteria (non-pathogen or pathogenic) either in monocultures or mixtures under common garden conditions that included herbivory. Specifically, we asked whether differential compositions of bacterial species in the phyllosphere would predict plant performance under these conditions. Moreover, we also asked how bacterial species richness and interspecific interactions structure the development and fitness of four genetically diverse Arabidopsis accessions.

## Materials and Methods

### Arabidopsis Accessions

We used four *Arabidopsis thaliana* accessions, Ba.1.2, Tu-0, NFA-8, and Kelst-4, which represented a broad geographic sampling from northern, western, and southern Europe (**Supplementary Figure S1**; Supplementary Table S1) (Atwell et al., 2010; Todesco et al., 2010). Moreover, according to the kinship matrix, these accessions are genetically different from each other (Supplementary Table S2), and can reach reproductive maturity successfully under our common-garden conditions.

### Bacterial Species

We used three bacteria species namely *Bacillus cereus* (non-pathogen), *X. campestris* (pathogen), and *P. syringae* (*Pst DC3000* pathogen) in this study. Unless otherwise stated in the manuscript, we refer to these three species by their commonly used names, *Pst DC3000, Xanthomonas*, and *Bacillus*, respectively. The bacterial strains *Bacillus*, and *Xanthomonas* were originally isolated from the Arabidopsis phyllosphere in a previous study (Traw et al., 2007) whereas the *Pst DC3000* is a model phytopathogen; all three bacterial species are widely used in studies of plant–microbe interactions (Kover and Schaal, 2002; Korves and Bergelson, 2003; Ji et al., 2014).

### Experimental Set Up and Design

We sowed seeds of all Arabidopsis accessions in wet Promix – BX (Premier Horticulture, Red Hill, PA, USA) in 36-celled flats. After a chilling treatment at 4°C in dark for 1 week, we transferred covered flats to the growth room at 20°C under a 12 h day with 55% humidity experiencing herbivory by the fungus gnats. We watered plant flats *ad libitum* from below using undertrays where the plants were fertilized two times with Peter’s 20:20:20 NPK solution (1.2 g per liter). Each cell received about 20 ml of fertilizer solution.

The bacterial strains were grown overnight in liquid KB media and then spun in the centrifuge (∼3,000 rpm, 5 min) to obtain pellets. The pellet was then resuspended in 10 mM MgSO_4_) and then standardized by spectrophotometer. We sprayed the bacterial suspensions (low concentration < 0.1OD_600__nm_) of the monocultures and mixtures on the different flats containing 2 weeks old seedlings, so that the Arabidopsis accessions would then establish their own respective microbial communities with time. We treated control plants with the 10 mM MgSO_4_ buffer only to control for the effect of spraying. Overall, the experiment comprised five treatments: (i) controls without bacterial application, (ii) three individual application of one out of the three bacterial species, and (iii) bacterial mixture application (all three bacterial species together). Unless otherwise stated in the manuscript, both bacterial treated and control treated plants were grown in the same growth room experiencing fungus gnat herbivory. In our experiment, we did not have control over herbivory and/or any herbivore free treatment. Thus, our focus is on the comparative effect of different bacterial species mixtures on plant performance in common garden conditions under herbivory.

During the application of bacterial monocultures and mixtures, we used the substitutive experimental design to determine the impact of bacterial species richness on bacterial abundance and plant performance (Saleem et al., 2016a). While our bacterial species diversity level was simple (monoculture vs. mixture of three species); nevertheless, it has sufficient range to provide valuable insights into microbial interactions and their impacts on the plant-microbe performance. Other studies have produced valuable information from experiments conducted with, similarly, low species diversity treatments (e.g., Gamfeldt et al., 2005). The growth room was equally invaded by the fungus gnat, thus representing near-natural conditions and plants were randomized within the room. However, we did not have any herbivore-free treatment and therefore did not specifically assess the effect of the insect on plant or bacterial performance. All known control methods of fungus gnats, including nematodes, have unacceptable side effects in that they upregulate plant immune responses (Jagdale et al., 2009). The position of plants in the growth room was changed every second day to minimize position effects.

### Data Measurement

At maturity (∼after 50 days), we sampled leaves from different flats to determine the abundance of bacteria in the monocultures and mixtures. Briefly, we selected randomly leaves for sampling and used a hole-punch to obtain a leaf disk (0.28 cm^2^), which was ground and mixed in 100 μL of buffer (10 mM MgSO_4_). The appropriate dilutions of sample mixtures were then plated on KB agar plates without antibiotic for *Bacillus* and *Xanthomonas* whereas the *Pst DC3000* plates contained 50 μg ml^-1^ of rifampicin. All plates were incubated at 28°C for 3 days before counting the number of colonies using a standard method (Ji et al., 2014). Meanwhile, we also recorded plant fitness data, such as days to flowering (DF), siliques per plant (SPP), seeds per silique (SPS), and plant biomass (aboveground only) depending on plant reproductive developmental stages. The above-ground plant biomass was freeze-dried to determine the dry biomass.

### Statistical Analysis

**FIGURE 1:**
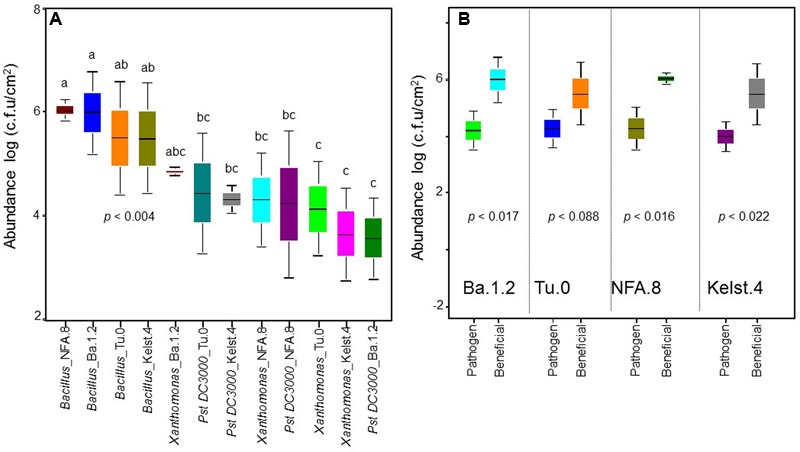
**Abundance of bacterial monocultures in the phyllosphere of Arabidopsis accessions.** The performance of bacterial monocultures in the phyllosphere of all Arabidopsis accessions **(A)**. The comparison between the performance of beneficial (*Bacillus*) and pathogenic (*Pst DC3000* and *Xanthomonas* together) bacteria in the phyllosphere of all Arabidopsis accessions **(B)**. The monoculture treatments are the average of three replicates, whereas the panel **(B)** shows an average of all replicates in the both beneficial and pathogenic treatments. The significant differences were determined by ANOVA followed by Tukey’s test. Error bars represent means ± 1SE. Lack of shared letters above the bars indicate significant differences.

**FIGURE 2:**
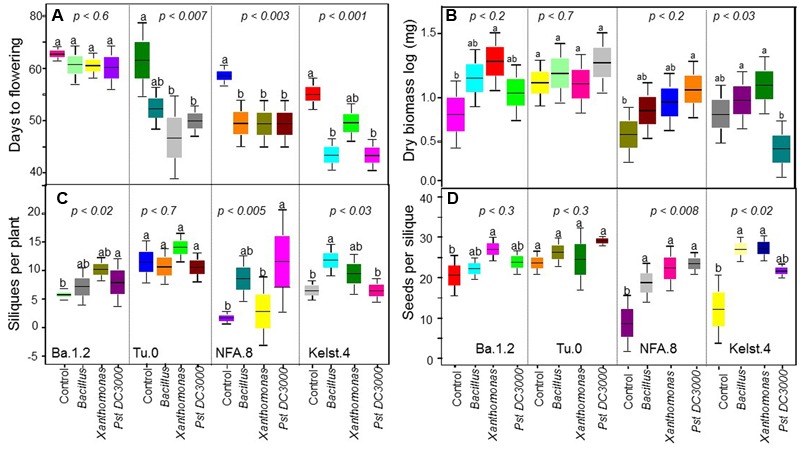
**Impact of individual bacterial species on the plant performance.** Effect of bacterial monoculture treatments on **(A)** days to flowering (DF), **(B)** dry biomass per plant, **(C)** siliques per plant (SPP), and **(D)** seeds per silique (SPS) production in all Arabidopsis accessions (NFA-8, Ba1-2, Kelst-4, and Tu-0). Sample size varied from 6 to 15 plants (e.g., Kover and Schaal, 2002). Error bars represent means ± 1SE. The significant differences were determined by ANOVA followed by Tukey’s, and Fisher’s tests. Lack of shared letters above the bars indicate significant difference.

**FIGURE 3:**
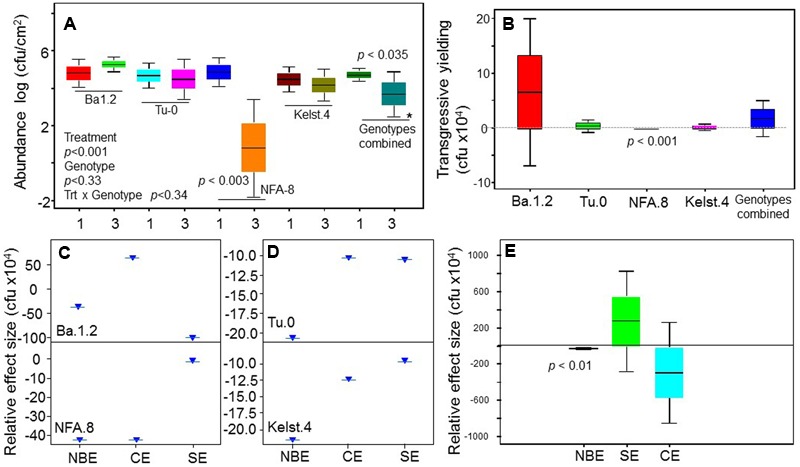
**Impact of bacterial species richness on the bacterial abundance, and the relative size of diversity effects in bacterial communities across plant accessions.** Effects of bacterial species richness on the bacterial abundance in the phyllosphere of all Arabidopsis accessions **(A)**. All monoculture and mixture treatments were in triplicate. Significant differences were determined by ANOVA followed by Tukey’s test. Error bars represent means ± 1SE. The tripartite equations were applied to calculate the **(B)** transgressive yield, **(C,D)** net biodiversity effect (NBE), complementarity effect (CE), and selection effect (SE) for each genotype, and **(E)** averaged diversity effects across all accessions.

All bacterial data (c.f.u/cm^2^) were normalized (+0.1), and log-transformed. To determine the significant differences in the bacterial abundance across various treatments, we used ANOVA followed by a Tukey-Test (**Figures 1–3A**). The bacterial abundance in the monocultures and mixtures was our metric for estimating biodiversity effects (Fox, 2005). To partition the bacterial species biodiversity effects, we applied Fox’s tripartite equations (Fox, 2005). Here, the three types of bacterial interspecific interactions whose combined effect is called as the net biodiversity effect (NBE) are called individually the dominance effect (DE), trait-dependent complementarity (TDC) and trait-independent complementarity (TIC) effects (**Figures 3C–E**). The NBE is the difference between the observed total yield and the expected total yield of a bacterial mixture assuming that inter and intraspecific interactions are same (null hypothesis). This equation is derived from the additive bi-partitioning equation that partitions NBE into the selection effect (SE) and complementarity effect (CE) (Loreau and Hector, 2001).

$$\Delta Y=N\bar{\Delta \text{RY}}\times \bar{M}+Ncov\left(M,{\text{RY}}_{O}-\frac{{\text{RY}}_{O}}{{\text{RYT}}_{O}}\right)+Ncov\left(M,\frac{{\text{RY}}_{O}}{{\text{RYT}}_{O}}-{\text{RY}}_{E}\right)$$

Δ*Y* = Net biodiversity effect (NBE)

*N* = Number of bacterial species in mixture


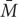
 = Average abundance of all bacterial species in monoculture

RY*_O_* = Observed yield of all bacterial species in the mixture, i.e., cell number of a bacterial species in the mixture divided by its cell number in monoculture.

RY*_E_* = Expected relative yield of all bacterial species in the mixture, i.e., the proportion in which a bacterial species was added to the mixture.


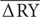
 = Average deviation between bacterial RY*_O_* and RY*_E_* in the mixture.

RYT*_O_* = Sum of RY*_O_* of all bacterial species in the mixture.

The first term on the right side of the equation (N
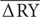
×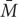) is the TIC that is equal to CE (Loreau and Hector, 2001). While the first covariance term in the above equation Ncov 
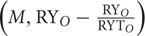
 is the TDC, whereas the last covariance term Ncov 
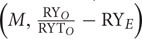
 is the DE. Sum of TDC and DE is equal to SE (Loreau and Hector, 2001).

**FIGURE 4:**
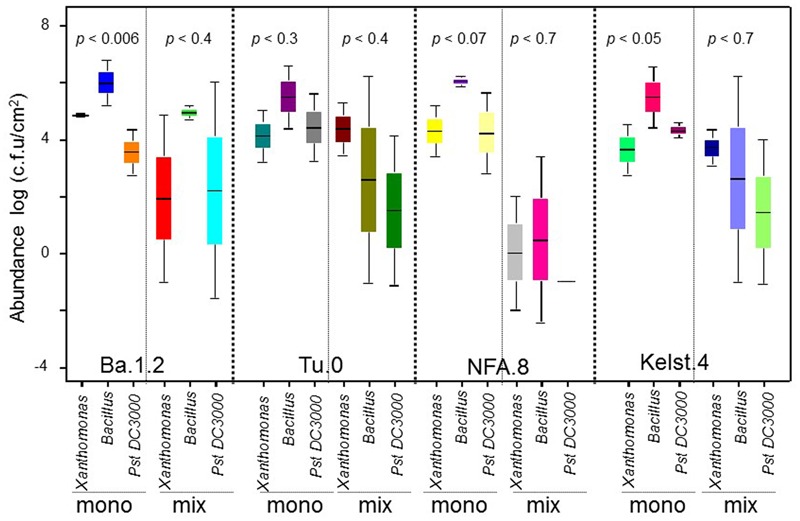
**A comparison of the performance of bacterial species in the monocultures and mixtures across all plant accessions.** The comparative performance of all bacterial species in the monoculture and mixture across all Arabidopsis accessions. All monoculture and mixture treatments were in triplicate. Significant differences were determined by ANOVA followed by Fisher’s test. Error bars represent means ± 1SE.

**FIGURE 5:**
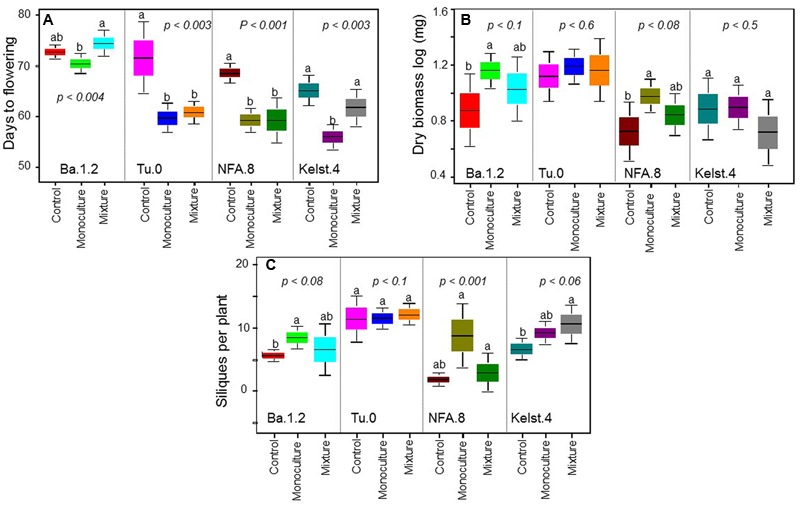
**Impact of bacterial species richness on plant performance.** Effect of bacterial species richness (monoculture vs mixture) on **(A)** DF, **(B)** dry biomass per plant, and **(C)** SPP production in all Arabidopsis accessions. Sample size varied from 6 to 15 plants (e.g., Kover and Schaal, 2002). Error bars represent means ± 1SE. The significant differences were determined by ANOVA followed by Fisher’s tests. Lack of shared letters above the bars indicate significant difference.

**FIGURE 6:**
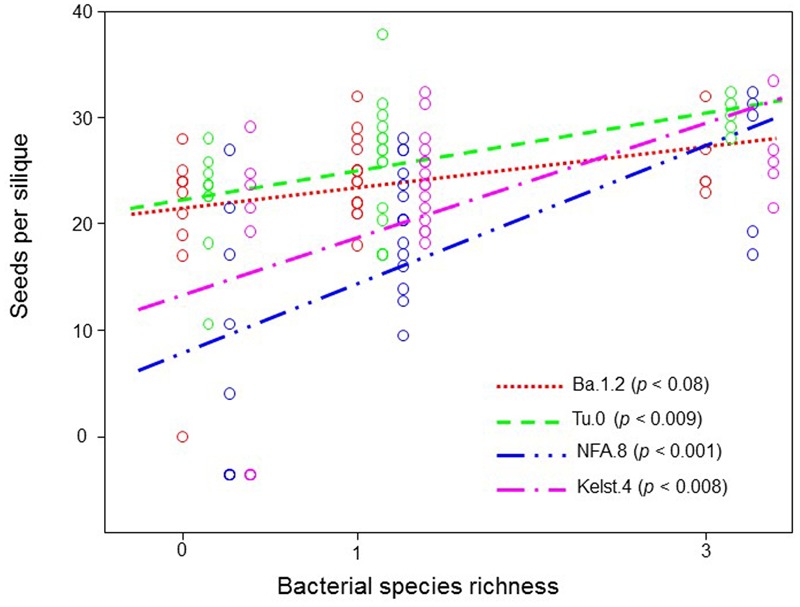
**Impact of bacterial species richness on the SPS production across all plant accessions.** Observed seed per silique production in all Arabidopsis accessions as a function of bacterial species richness across all treatments. Each point in the figure corresponds to the average of various replicates (mostly > 3). The points mentioning to respective treatments at equal richness level are slightly offset horizontally for clarity. The ANOVA with a linear fitting on the means was performed to determine the effect of bacterial species richness on SPS.

To determine significant differences in plant performance across various treatments, we used ANOVA followed by Tukey’s and Fisher’s tests (**Figures 1–5**). To assess the relationship between the bacteria species richness and SPS production, we used general linear regression followed by ANOVA (**Figure 6**).

## Results

### Abundance of Individual Bacterial Species in the Phyllosphere under Herbivory

The abundance of bacterial species varied significantly in the phyllosphere of all Arabidopsis accessions measured under common garden conditions (**Figure 1A**). The beneficial bacteria *Bacillus* showed a relatively greater abundance in the phyllosphere, irrespective of host plant identity. Contrarily, both *Xanthomonas* and *Pst DC3000* (**Figure 1A**) showed a relatively lower abundance in the Arabidopsis accessions. Overall, we observed the highest (*Bacillus*) and lowest (*Pst DC3000*) bacterial abundances in the phyllosphere of NFA.8 and Ba.1.2 accessions, respectively. To get a clearer picture of the data, we compared the abundance of *Bacillus, Xanthomonas*, and *Pst DC3000* (**Figure 1B**). Collectively, all Arabidopsis accessions showed a relatively higher abundance of beneficial than pathogenic bacteria in the phyllosphere (**Figures 1B** and **4**).

### Impact of Individual Bacterial Species on Plant Performance under Herbivory

The Arabidopsis accessions responded differentially to various bacterial treatments in the expression of their fitness traits (i.e., DF, plant biomass, SPP, and SPS) (**Figure 2**). Three accessions, Tu.0, NFA.8, and Kelst.4, all responded significantly in their flowering phenology to bacterial inoculation, by flowering earlier than control plants. In contrast, the Ba.1.2 accession did not differ statistically in flowering earliness (**Figure 2A**). Among bacteria, time to flowering was significantly reduced by *Xanthomonas* in the Tu.0 plants. *Pst DC3000* and *Bacillus* both promoted early flowering in inoculated plants relative to controls in the Kelst.4 accession (**Figure 2A**). Similarly, all bacterial species substantially influenced plant biomass. Plants treated with the monoculture bacterial treatments yielded more biomass than the control plants (**Figure 2B**). *Xanthomonas* and *Pst DC3000* had the strongest influence on the biomass of their host plants under these common garden conditions (**Figure 2B**). The Arabidopsis accessions NFA.8 and Kelst.4, when grown under the influence of *Pst DC3000*, produced relatively greater and lower biomass than the control plants, respectively. The *Xanthomonas* treatment significantly increased biomass production in Ba1.2 than the control plants (**Figure 2B**). Mostly the bacterial treated plants produced relatively more seeds per plant than the control plants. Overall, except in Tu.0, bacterial treatment significantly altered the silique production in the Arabidopsis accessions (**Figure 2C**). Specifically, the Arabidopsis accessions, such as NFA.8 and Kelst.4 produced relatively more seeds per plant under the influence of *Pst DC3000* and *Bacillus*, respectively, than in the controls. Furthermore, both pathogenic and beneficial bacteria also influenced the number of SPS in all four Arabidopsis accessions (**Figure 2D**). Overall, the bacterial treated plants produced substantially more SPS than control plants, most significantly for the accessions NFA.8 and Kelst.4 (**Figure 2D**). For the Ba.1.2 accession, seed number per silique was increased only under the influence of *Xanthomonas* (**Figure 2D**).

### Impact of Bacterial Species Richness on Bacterial Abundance in the Phyllosphere under Herbivory

Bacterial concentrations differed markedly in the phyllosphere of the Arabidopsis accessions, particularly in the monoculture treatments (**Figure 4**). Mostly, bacterial species richness negatively affected bacterial abundance, indicating negative bacterial diversity effects. Bacterial abundance was significantly lower in mixture than monocultures in the NFA.8 accession (**Figure 3A**), and resultantly, the bacterial mixtures demonstrated a consistent transgressive underyielding in all Arabidopsis accessions (**Figure 3B**). The mixture yield was significantly below zero in the NFA.8 plants. Using the Fox’s tripartite biodiversity equations derived from the Loreau and Hector (2001) bipartite model, we determined why and how the bacterial mixture performed poorly. These questions helped us to identify the relative effect sizes of diversity effects on bacterial abundance in the phyllosphere of all accessions. These equations partitioned the bacterial diversity effects into the CE, and the SE. The composite response of CE and SE is called as the NBE (Loreau and Hector, 2001). The positive CE implies facilitative or positive interactions (niche partitioning) while the negative SE reflects chemical interference (or antagonistic interactions) among species in the mixture. The positive and negative SE implies the selection process that favors some productive species in the mixture due to their certain traits or the dominance. The NBE was negative in all cases (**Figures 3C–E**), and on average it differed statistically from zero (**Figure 3E**). Therefore, the relative effect sizes of the NBE components such as the CE, and SE were weak and varied in all Arabidopsis accessions (**Figures 3C,D**). The positive CE observed in the bacterial communities of Ba.1.2 could be due to the relatively better or same performances of some species (*Pst DC3000* and *Xanthomonas*) in the mixture (**Figure 4**). The negative CE and SE clearly reflected antagonistic interactions among the bacterial species (**Figures 3C,D**) that ultimately resulted in an overall poor performance of the bacterial species in the mixtures across all Arabidopsis accessions (**Figures 3A** and **4**).

### Impact of Bacterial Species Richness on Plant Performance

Exposure of plants to bacterial mixtures delayed flowering in these plants relative to the monoculture treated plants (**Figure 5A**). The Arabidopsis accession, Ba.1.2 when grown under the influence of bacterial mixture, flowered later than the control plants. All other Arabidopsis accessions growing under the bacterial influence flowered earlier than the control plants (**Figure 5A**). Corresponding to the poor performance (abundance) of bacterial mixtures, interestingly all Arabidopsis accessions produced relatively less biomass under the bacterial mixtures than under the monocultures (**Figure 5B**). Except Kelst.4, the plants grown under the influence of bacterial mixtures produced relatively more biomass than control plants. As mentioned, the Kelst.4 plants produced less biomass in the bacterial mixture than controls. Similarly, bacterial species richness differentially impacted the seeds per plant in the host Arabidopsis accessions under these conditions. Similar to the biomass production, most of the cases, the bacterial mixtures had poor impact on seeds per plant than that of the bacterial monocultures (**Figure 5C**). Contrarily, bacterial species richness tended to increase seed production in the Arabidopsis population, Kelst.4 (**Figure 5C**). Very interestingly, the seed number per silique increased linearly and significantly across bacterial species richness, irrespective of the Arabidopsis accessions (**Figure 6**). Overall, the total seed production per plant also increased linearly with the bacterial species richness with some variation (**Supplementary Figure S2**).

## Discussion

Insects and pathogens are among the factors most limiting to crop production. Therefore, research on the PMI interactions is instrumental to understanding the plant responses to natural enemies in a broader agroecological context. Here, we report the impacts of phyllosphere–bacterial interactions on plant performance, determined here from assessments of the plant–microbe interaction across the plant life cycle. The phyllosphere supported a relatively greater abundance of beneficial rather than pathogenic bacteria under our common garden conditions that included the presence of fungus gnat herbivory (**Figures 1A,B** and **4**), which is consistent with the view that hosts recruit beneficial microbes in relatively greater numbers under stressed and normal conditions to obtain the maximum mutualistic benefits (Vorholt, 2012; Ortega et al., 2016; Saleem et al., 2016b). The *Bacillus* species group is ubiquitously abundant in soil and plant environments with multiple plant beneficial (anti-insect/pathogen) properties. Partly similar to our findings, phyllosphere ecosystems showed a significant presence of *Bacillus* under insect and pathogen attacks in a few previous studies (Jacobsen et al., 2004; Collier et al., 2005). Contrarily, a reduced abundance of the pathogens reflected host resistance against the pathogens that arises when the natural enemies (insects, pathogens) induce host defense against each other (antagonism among natural enemies) (Rayapuram et al., 2008). These results differ from a recent study in which a greater abundance of the *Pst DC3000* in the phyllosphere of a mustard, *Cardamine cordifolia* (Brassicaceae), was attributed to the insect herbivory (synergism among natural enemies) (Humphrey et al., 2014).

Contrary to the abundance trend, plants grown with the beneficial bacteria (*Bacillus*) performed relatively poorly in their growth, developmental and reproductive traits such as the DF, plant biomass, SPP and SPS production than with the pathogenic bacteria (*Pseudomonas* and *Xanthomonas*) under herbivory (**Figure 2**). Moreover, similar to some previous short-term studies, plants did not show disease symptoms under the influence of pathogens alone or with insect herbivores (e.g., Kover and Schaal, 2002; Korves and Bergelson, 2003; Vos et al., 2006). The pathogen-induced early flowering and consistently greater plant performance results that we observed are in line with evolutionary models emphasizing that plants challenged by natural enemies often develop and reproduce more quickly (Korves and Bergelson, 2003). However, flowering time may depend in part on host tolerance and invader species, in the sense that non-tolerant host plants experiencing herbivore or pathogen stress may flower late due to their retarded acquisition of resources (Elzinga et al., 2007). A greater plant biomass and corresponding better reproductive performance may reflect the influence of microbes in altering the nutritional quality of plants to minimize the herbivore damage (antagonism among natural enemies). However, the opposite seems to be true for plant biomass production in our study, which was reduced for example in the Kelst.4 accession under exposure to *Pst DC3000*. This is consistant with synergism among natural enemies (Stout et al., 1999; Biere and Bennett, 2013), and suggests the significance of plant genotype identity in structuring PMI interactions. The better expression of some plant traits with *Bacillus* than with other treatments (control or pathogen), is not surprising since these bacteria may promote plant growth through various mechanisms (i.e., production of foliar primary and secondary metabolites) under herbivory (Gange et al., 2012). Overall, the plant responses to various bacterial species differed substantially either in the form of phyllosphere bacterial abundance or plant trait expressions that highlight the importance of host genetic variations in shaping PMI interactions (Kover and Schaal, 2002). Despite these interpretations, there is still a great need to understand the mechanisms of microbial recruitment by plant leaves, and their respective impacts on host plant fitness under a broad range of environmental conditions.

Our finding that bacterial mixtures performed poorly in the phyllosphere of all four Arabidopsis accessions is notable. Our results revealed a negative effect of bacterial species richness on bacterial abundance (**Figure 3A**) in a way that bacterial mixtures demonstrated a consistent transgressive underyielding. We observed negative NBEs in most of the cases with weak complementarity and SEs (**Figures 3C–E**). Such weak negative and positive biodiversity (CE, SE) effects have been reported in prior microbial model system studies (Jiang, 2007; Becker et al., 2012; Saleem et al., 2012), thus suggesting that neither of these bacteria competitively excluded other species from the phyllosphere community. It is very likely that the bacterial mixtures performed poorly due to particular growth-limiting conditions, such as light, moisture, and nutrient stress, etc.) (Lindow and Brandl, 2003; Meyer and Leveau, 2011; Vorholt, 2012; Saleem et al., 2015) and antagonistic interactions (Maida et al., 2016) in the phyllosphere. Though bacterial mixtures exhibited a differential growth, they performed much poorer in the phyllosphere of NFA.8 plants for which one (*Pst DC3000*) out of three bacterial species was absent in the mixture. The reduced bacterial growth in NFA.8 plants points to the more likely influence of host genetic variations on the phyllosphere colonizers (Kover and Schaal, 2002).

Previously, negative biodiversity effects are rarely linked directly to the microbial-driven host phenotypes (but see, Becker et al., 2012). Jiang et al. (2008) postulated that poor or negative biodiversity effects (CE, SE) “may be potentially common for non-biomass functions” for which the species abundance in number (**Figures 5** and **6**) is likely a poor predictor of functional impact. Our results thus confirmed previous theoretical predictions (Jiang et al., 2008) showing weak negative biodiversity effects influenced key parameters of plant performance (**Figures 5** and **6**).

The bacterial species richness, similar to its influence on bacterial abundance, mostly did not increase plant performance (plant biomass, SPP, flowering time) than bacterial monocultures. However, our results revealed a positive impact of bacterial species richness on the SPS production (**Figure 6**) that are in line with the biodiversity-functioning theory stating that relatively diverse microbial communities and trophic interactions ensure greater ecosystem services (e.g., seed production) (Saleem and Moe, 2014; Saleem, 2015a). Moreover, we also for the first time show that weak microbial biodiversity effects could potentially influence host reproductive fitness (Jiang et al., 2008). However, at this point, these results are not conclusive and require further tests to a full understanding of the role of higher microbial biodiversity in the expression of host developmental and reproductive traits. Overall, our findings of improved plant performance under bacterial influence (monoculture or mixture pathogen species) also confirmed a recent theoretical prediction stating that combinations of multiple enemies (herbivores and pathogens together) cause less reduction in the plant performance than individual enemies in isolation (Stephens et al., 2013).

Given that the PMI interactions are complex, there are at least three plausible non-exclusive explanations why plants performed well under our common garden conditions of insect herbivory stress; (i) both types of bacteria may have induced systemic resistance in the plants against insect herbivores by modulating the defense signaling molecules (Sugio et al., 2014), (ii) antagonistic interactions may have occurred among multiple natural enemies (Stephens et al., 2013), and (iii) differential response of the Arabidopsis accessions to natural enemies may have been structured by their genetic and demographic history (Korves and Bergelson, 2003; Züst et al., 2011).

## Conclusion

The improved plant performance that we report here in responses to the beneficial or pathogenic bacteria or their combination reflects the importance of preserving ecological diversity in balancing the impact of natural enemies on host plants. Our finding that the bacterial species richness and antagonistic interactions regulate host development and fitness, is a significant advance in the mechanistic understanding of the plant–microbe interactions under herbivory. Therefore, in future studies, dissecting the relative significance of environmental stressors in the plant performance is critical to predicting the consequences of land use and global change because plants, microbes, and insect herbivores are highly sensitive to this change.

## Author Contributions

MS and MT designed the study. MS, NM, ZP, and MT collected phenotypes. MS and MT carried out the analysis. MS and MT wrote the paper. All authors discussed the results and commented on the manuscript.

## Conflict of Interest Statement

The authors declare that the research was conducted in the absence of any commercial or financial relationships that could be construed as a potential conflict of interest.

The reviewer AA and handling Editor declared their shared affiliation, and the handling Editor states that the process nevertheless met the standards of a fair and objective review.

## Supplementary Material

FIGURE S1 **Map showing source accessions for the four genotypes studied.** Site details are shown in Supplementary Table S1.

FIGURE S2 **Effects of bacterial species richness on plant total seed production in all accessions.** The species richness levels 0, 1, 3 correspond to control, bacterial monoculture, and mixture treatments.
