# Supplementary figures and images for: Microbial Interactions in the Phyllosphere Increase Plant Performance under Herbivore Biotic Stress
Source: Front Microbiol. 2017 Jan 20;8:41. doi: 10.3389/fmicb.2017.00041 (PMC5247453; doi:10.3389/fmicb.2017.00041)

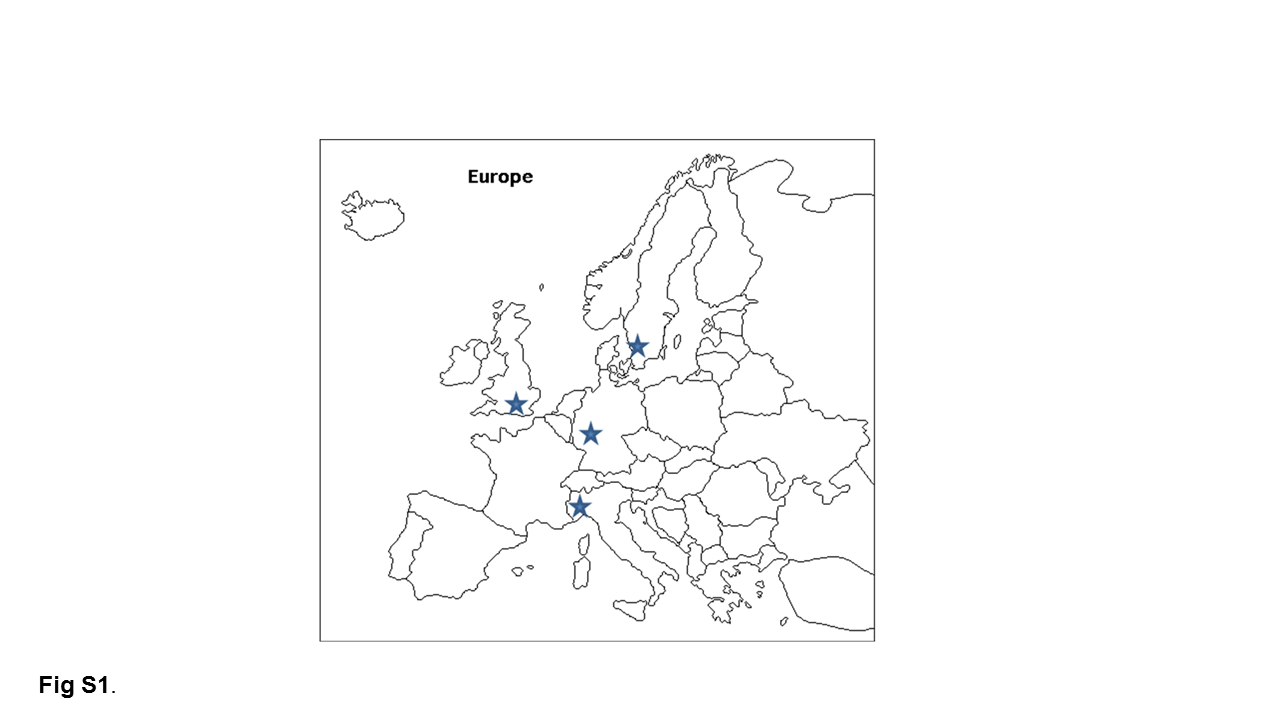

Supplement: FIGURE S1 — Map showing source accessions for the four genotypes studied. Site details are shown in Supplementary Table S1. [file Image_1.TIF]

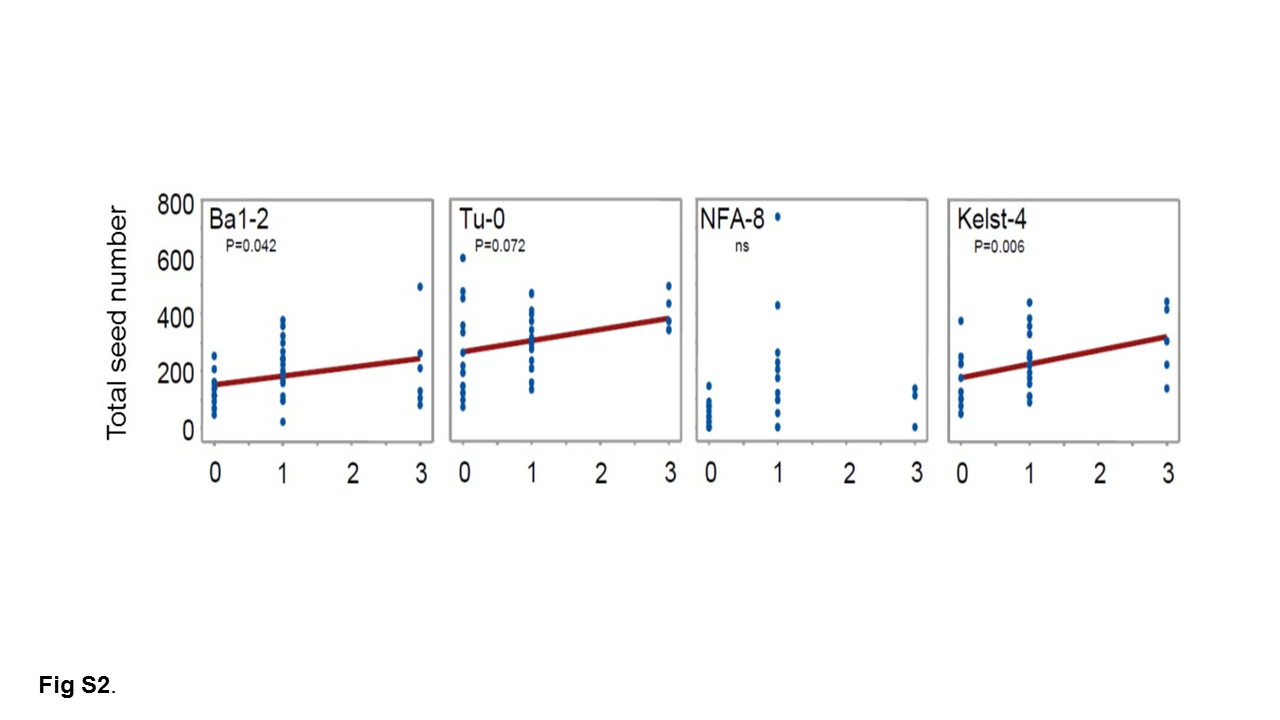

Supplement: FIGURE S2 — Effects of bacterial species richness on plant total seed production in all accessions. The species richness levels 0, 1, 3 correspond to control, bacterial monoculture, and mixture treatments. [file Image_2.TIF]
