# Supplementary material for: Microbial Interactions in the Phyllosphere Increase Plant Performance under Herbivore Biotic Stress
Source: Front Microbiol. 2017 Jan 20;8:41. doi: 10.3389/fmicb.2017.00041 (PMC5247453; doi:10.3389/fmicb.2017.00041)
Supplement: Supplementary file 3 [file Table_1.PDF]

Table S1. Latitude and longitude coordinates of the source populations for each of the four genotypes.

Genotype Name	Ecotype No.	Accession No.	Latitude	Longitude	Site	Country	Region
Bå1-2	8256	CS76676	56.4	12.9	Båstad	SWE	N Europe
Kelsterbach-4	8420	CS6041	50.0667	8.5333	Kelsterbach	GER	W Europe
NFA-8	6944	CS22598	51.4083	-0.6383	Silwood Park	UK	N Europe
Tu-0	8395	CS1566	45	7.5	Turin	ITA	S Europe
