# Supplementary material for: Microbial Interactions in the Phyllosphere Increase Plant Performance under Herbivore Biotic Stress
Source: Front Microbiol. 2017 Jan 20;8:41. doi: 10.3389/fmicb.2017.00041 (PMC5247453; doi:10.3389/fmicb.2017.00041)
Supplement: Supplementary file 4 [file Table_2.PDF]

Table S2. Kinship matrix (KM) of arabidopsis genotypes in this study. It tells us how to extent different genotypes are genetically related to each other. The largest value is 1 that represents 100% genetic similarity.

	Ba.1.2	Tu.0	NFA.8	Kelsterbach.4
Ba.1.2		0.653	0.646	0.671
Tu.0			0.63	0.683
NFA.8				0.643
